# Exploring associations between prenatal solvent exposures and teenage drug and alcohol use: a retrospective cohort study

**DOI:** 10.1186/s12940-017-0232-6

**Published:** 2017-03-11

**Authors:** Lisa G. Gallagher, Thomas F. Webster, Ann Aschengrau

**Affiliations:** 1 Department of Epidemiology, Boston University School of Public Health, 715 Albany Street T3E, Boston, MA 02118 USA; 2 Department of Environmental Health, Boston University School of Public Health, Boston, MA USA

**Keywords:** Tetrachloroethylene, Alcohol, Cohort, Drugs

## Abstract

**Background:**

Investigating the effects of prenatal and childhood exposures on behavioral health outcomes in adolescence is challenging given the lengthy period between the exposure and outcomes. We conducted a retrospective cohort study in Cape Cod, Massachusetts to evaluate the impact of prenatal and early childhood exposure to tetrachloroethylene (PCE)-contaminated drinking water on the occurrence of risk-taking behaviors as a teenager. An increased occurrence of risk-taking behaviors, particularly illicit drug use, was observed in those highly exposed to PCE. We hypothesized that there may be other sources of prenatal solvent exposure such as maternal consumption of alcoholic beverages during pregnancy which might modify the previously observed associations between PCE and risk-taking behaviors and so we conducted an exploratory analysis using available cohort data. The current report presents the results of these analyses and describes the difficulties in conducting research on long-term behavioral effects of early life exposures.

**Methods:**

The exploratory analysis compared a referent group of subjects with no early life exposure to PCE or alcohol (*n* = 242) to subjects with only alcohol exposure (*n* = 201), subjects with only PCE exposure (*n* = 361), and subjects with exposure to both PCE and alcohol (*n* = 302). Surveys completed by the subject’s mother included questions on prenatal alcoholic beverage consumption and available confounding variables such as cigarette smoking and marijuana use. Surveys completed by the subjects included questions on risk-taking behaviors such as alcoholic beverage consumption and illicit drug use as a teenager and available confounding variables. PCE exposure was modeled using a leaching and transport algorithm embedded in water distribution system modeling software that estimated the amount of PCE delivered to a subject’s residence during gestation and early childhood.

**Results:**

Subjects with early life exposure to both PCE and alcohol had an increased risk of using two or more major drugs as a teen (RR = 1.9 (95% CI 1.2, 3.0)) compared to unexposed subjects. Increased risks for only PCE exposure (RR = 1.6 (95% CI 1.0, 2.4) and only alcohol exposure (RR = 1.3 (95% CI 0.7, 2.1)) were also evident but were smaller than the increased risk associated with both exposures. While available confounding variables were controlled, many relevant social risk factors were not obtained due to limitations in the retrospective study design.

**Conclusions:**

This exploratory analysis found evidence for an additive effect of early life exposure to PCE and alcohol on the risk of use of multiple illicit drugs as a teenager. Because of numerous limitations in this retrospective study, further research is needed to examine longstanding behavioral effects of early life exposures. To be most informative, this research should involve long-term prospective data collection.

## Background

Investigating the effects of prenatal and childhood exposures on behavioral health outcomes in adolescence is challenging for many reasons. Given the lengthy period between the exposure and outcomes, accurate data on confounding and mediating factors may be difficult to obtain. We previously examined early life exposure to tetrachloroethylene (PCE)-contaminated drinking water and teen risk-taking behaviors, specifically cigarette smoking, alcohol and illicit drug use, in a retrospective cohort study in the Cape Cod region of Massachusetts [1]. Exposure to PCE-contaminated water occurred when PCE leached from a vinyl-liner applied to the inner surface of water distribution pipes to prevent taste and odor problems. Because the vinyl-lined pipes were used to replace or expand the existing distribution system [2], the irregular contamination pattern led to neighbors with very different exposure levels.

Our prior study found an increase in risk-taking behaviors to be associated with high PCE exposure in early life while controlling for available confounding variables [1]. These results are supported by a recent review noting neurotoxicity as a sensitive outcome of PCE exposure [3]. In particular, occupational studies of PCE and other solvents among adults have observed impaired cognition, memory, attention and executive function as well as associations with mood and behavior [4–11]. Some studies have also shown that early life exposures to solvents can negatively impact neuropsychological test scores and behavior among children [12, 13].

Prenatal exposure to another organic solvent –alcohol-- has also shown negative impacts on behavior. In particular, increases in risk of alcohol disorders later in life have been observed, independent of other intervening factors such as maternal smoking and drinking during childhood [14, 15]. Animal and human data support the hypothesis that prenatal alcohol exposure increases affinity for later use [16], and alcohol exposure during pregnancy is known to have adverse effects on brain development [17]. In particular, fetal alcohol spectrum disorders (FASD), a condition resulting from high levels of maternal alcohol consumption, ranges from severe effects such as facial anomalies and growth retardation to mild and moderate effects on cognition and behavior, including impacts on executive functioning and motor control [18]. Less is known about the long-term behavioral effects of light and moderate drinking during pregnancy as well as the combined impact with other exposures during this period [17].

Given the previously observed associations observed for teenage risk-taking behaviors with early life exposure to PCE in our study and with alcohol in others, we conducted exploratory analyses to assess the neurotoxic effects of these common and concurrent exposures in pregnant women on their children later in life. Existing data on maternal alcohol consumption were available in our historical birth cohort (from 1970s through early 1980s) and observed at a higher prevalence than today because the harmful effects of prenatal alcohol consumption were less well known.

We hypothesized that each of these exposures would negatively impact neurodevelopment and would be associated with increases in teenage alcohol and drug use, and that their combined exposure would produce an effect equal to or greater than the sum of effects for each exposure alone. Possible biological mechanisms for these effects involve impaired impulse control and judgement, increased affinity for these substances, and greater consumption requirements to achieve the desired effect on mood and behavior [16, 19]. The current report presents the results of these exploratory analyses and describes the numerous challenges of conducting research on long-term behavioral effects of early life exposures.

## Methods

### Study population

The study population was identified by reviewing all the births to married women between 1969 and 1983 in the eight towns in the Cape Cod region of Massachusetts that were known to contain some vinyl-lined water distribution pipes (Barnstable, Bourne, Brewster, Chatham, Falmouth, Mashpee, Provincetown and Sandwich). The maternal address and birth year on each birth certificate were compared to known locations and installation years of VL/AC pipes. Subjects were initially designated as exposed to PCE if their residence at birth was directly adjacent to a VL/AC pipe that was installed prior to birth or if the only possible water flow to their residence was through a VL/AC pipe (*n* = 1,910). Comparable unexposed subjects (*n* = 1,928) were randomly selected from the remaining births, using frequency matching by month and year of birth. These subjects were initially considered “unexposed” due to the larger distance from their residence to the VL/AC pipe. Older siblings of the identified subjects born during the study period were added to the study population (*n* = 1,202). These subjects were initially considered unexposed at birth. More detailed assessments of water flow were later completed to more accurately assess exposure of all subjects.

In addition to the maternal address, each subject’s name, parents’ names, subject’s date of birth, birth weight and gestational duration, and parents’ names, ages and educational levels were also gathered from the birth certificate.

### Data collection

Subjects were located and enrolled between 2006 and 2008. The current address and telephone number of each subject were obtained using Massachusetts Residents’ Lists; death, credit bureau, and alumni records; and Internet and print telephone listings. Subjects were contacted by mail with a description of the study and a request to fill out a self-administered questionnaire. Subjects who did not respond were contacted by letter up to three times and then by telephone when possible.

As described in more detail in a prior report, subjects were excluded during enrollment because they were either deceased, never located, refused, or never responded or their parent refused participation in our prior study [1]. Questionnaires were returned by 33.5% of the selected subjects although this percentage of participants was slightly higher (40.5%) when based on subjects who could be located. Inadequate residential histories and residence in a town off-Cape with unquantified VL/AC exposure excluded another 10% of the subjects. Detailed exposure assessments did identify changes to some of the initial exposure designations.

A comparison of participants and non-participants based on birth certificate information indicated that they had similar PCE exposure status, race, age, birth order, mean gestational duration and birth weight. Participants were more likely than non-participants to be female and have college-educated mothers, but this was evident among exposed and unexposed subjects. More detailed information is provided in our prior report [1].

The self-administered questionnaire collected information on teenage smoking, alcohol and illicit drug use, as well as demographic characteristics. Subjects were asked if they ever consumed an alcoholic drink which was defined as a 12 oz bottle, can or glass of beer, 12 oz wine cooler, hard lemonade or hard cider, a 4 oz glass of wine, or a shot of liquor (straight or in a mixed drink). Additional details collected were the age at which they had their first drink and typical drinking frequency between the ages of 13 and 18. Teenage illicit drug use was characterized as ever use of marijuana, inhalants, heroin, cocaine or crack, psychedelics or hallucinogens, Ritalin without a prescription, and club drugs. Other relevant information obtained included race, ethnicity, marital status, education, occupation, personal and family history of learning disabilities and mental disorders, and other occupational and non-occupational sources of solvent exposures. Subjects were also asked about their personal knowledge of the PCE leaching incident and their exposure status. Birth address information was available on the birth certificate and confirmed by collection of a residential history of Cape Cod addresses from birth throughout childhood.

### Exposure assessment

Exposure to PCE was determined by modeling water flow through the VL/AC pipes to a given residence. First, Geographic Information Systems software (ArcGIS 8.1) was used to geocode residential birth addresses on Cape Cod to a latitude and longitude location. Residences were mainly located directly to their corresponding parcel of land using assessors’ files, county deeds, town voter lists and Internet resources. When this was not possible, residential addresses were either located (1) to the nearest possible parcel by street number or (2) if there was no street number, to the middle of the street if the street was less than one mile long, or finally (3) the intersection with the cross-street as provided in the survey if the street at least one mile long. Exposure and outcome status was unknown to the research staff conducting the geocoding who were able to successfully locate 95% of the addresses.

Visual inspection of the piping maps determined initial exposure status and was followed by a more detailed assessment using a leaching and transport model developed by Webler and Brown [20]. The model estimates the amount of PCE entering a residence by using the initial amount of PCE in the liner, the age of the pipe, and the leaching rate of PCE from the liner into the water. This rate is modeled as an exponential relationship with a rate constant of 2.25 years based on experimental data [21]. Additional model parameters include the water flow rate and direction which were determined using EPANET 2.0, water distribution system modeling software developed by the U.S. EPA and used in other epidemiological studies of drinking water contaminants [22–25]. The publically available “open-source” software code enabled direct incorporation of the Webler and Brown leaching algorithm into the simulation of water flow through the modeled pipe network. We developed schematics of each water system using historical information sources and assumed no appreciable changes occurred over the study period. We then examined each birth residence in our cohort and estimated an annual exposure for these locations, since only move-in year and installation year were available. The annual exposure was converted to represent the prenatal period (9/12 months) and assessed for all eligible subjects. Early life exposure was estimated as a cumulative measure by summing the estimated dose of PCE that entered their residences from the month and year following birth through the month and year of the fifth birthday. Partial years were accounted for using simple percentages of the annual measure.

Prenatal exposure to alcohol was collected by self-report from the subject’s mother who participated in an earlier study of the cohort. In particular, mothers were asked if they ever consumed alcoholic beverages during their first, second and third trimesters, and, if yes, they were asked about the frequency of consumption in each trimester [26]. Offspring were then divided into four groups based on their early life exposure to PCE and alcohol: (1) never exposed to PCE and never exposed to prenatal alcohol (2) never exposed to PCE and ever exposed to prenatal alcohol (3) ever exposed to PCE and never exposed to prenatal alcohol and (4) ever exposed to PCE and ever exposed to prenatal alcohol.

### Statistical analysis

The referent group for this analysis was comprised of individuals who were never exposed to PCE and never exposed to prenatal alcohol. The other exposure groups were compared to this unexposed group for the following outcomes: age at first drink (≤13 years vs. 14+ years), teenage drinking frequency (drank >8 days/month as teen vs. never drank as teen) and drinking amount (drank ≥5/4 drinks/day as a teen vs. never drank as a teen). We also evaluated use of specific drugs, any drugs, two or more drugs, any major drugs (excluding marijuana), and two or more major drugs and specific drugs compared to never using drugs.

The strength of the association between the combined PCE-alcohol exposure category and each outcome of interest was estimated using the risk ratio (RR). Ninety-five percent confidence intervals were used to assess the precision of the RRs. We performed both crude analyses and generalized estimating equation (GEE) analyses to account for non-independent outcomes arising from several children from the same family [27, 28]. Adjusted GEE analyses were conducted to assess confounding variables including demographic characteristics, factors associated with risk-taking behavior and prenatal alcohol consumption, and other sources of solvent exposure. These factors included the subject’s gender, race, age when the study questionnaire was completed, age at onset of puberty, breastfeeding status, and history of learning disabilities and mental illness. Parental information at the time of the subject’s birth included the mother’s age, educational level and history of mental illness; paternal age, educational level, occupation and history of mental illness; the mother’s prenatal care, multivitamin use, cigarette smoking, marijuana use, medical conditions, and obstetrical complications when she was pregnant with the subject; and number of prior live-born siblings. Childhood factors were limited but included the death of a sibling and maternal history of solvent exposure.

Each of these factors was compared by PCE exposure status and prenatal alcohol exposure status to evaluate if there was a greater than 5% difference between the exposure groups. As in our prior analysis, categorical variables were retained for consideration in the multivariate model if there was more than a 5% difference in the frequency of a characteristic between compared groups. For analyses involving both PCE and alcohol exposure, maternal educational level and paternal occupation at the subject’s birth, and number of prior live-born siblings met this criterion. Additionally, the subject’s breastfeeding status met this criterion for analyses involving PCE exposure alone, and the subject’s gender, age when the study questionnaire was completed, mother’s age, paternal age, mother’s cigarette smoking and mother’s marijuana use met this criterion for analyses of prenatal alcohol exposure alone.

These variables were added one at time to the multivariate model but none were retained because they did not change the crude GEE estimate by more than 10%. The one exception occurred when we adjusted for maternal cigarette smoking in the analysis for major drug use. Only one of the exposure category estimates (no PCE exposure and any alcohol exposure) was changed by more than 10% when maternal smoking was controlled (from 1.3 to 1.1). Changes to the estimates for the other exposure categories were less than 10% and thus the variable was not included in the final model. We have presented only unadjusted estimates as final analyses.

## Results

As previously described, subjects who met eligibility requirements were assessed for PCE exposure using a detailed water distribution model. Final exposure assessments determined 3% of subjects initially designated as exposed were unexposed and 33.7% of subjects initially designated as unexposed were exposed. After these changes, there were 831 subjects with prenatal and early life exposure to PCE and 547 subjects with no exposure that were available for analysis. Among these individuals, there were 168 PCE- exposed (20.2%) and 104 PCE- unexposed subjects (19.0%) who were missing prenatal alcohol exposure and were excluded from final analyses. We determined there were 302 subjects with exposure to both PCE and alcohol, 361 with only PCE exposure, 201 with only prenatal alcohol exposure and 242 subjects with neither exposure.

Comparison of the demographic characteristics revealed few differences by combined exposure status (Table 1). However, 52% of mothers who drank during pregnancy had graduated from college, compared to the 36–40% of mothers who did not drink. Consumption did tend to be low, 1–3 drinks per month, for the majority of mothers (62–64%). The majority of women who ever drank during pregnancy were also more likely to smoke cigarettes and marijuana but in both instances, frequencies of these behaviors were low.

**Table 1:** Distribution of Selected Subject Characteristics by Exposure Status^a^

Characteristic	Any PCE and any prenatal alcohol exposure (*N* = 302)		Any PCE and no prenatal alcohol exposure (*N* = 361)		No PCE and any prenatal alcohol exposure (*N* = 201)		No PCE and no prenatal alcohol exposure (*N* = 242)	
	*n*	%	*n*	%	*n*	%	*n*	%
Year of birth								
1969–1974	64	21.2	66	18.3	57	28.4	52	21.5
1975–1980	163	54.0	174	48.2	105	52.2	124	51.2
1981–1983	75	24.8	121	33.5	39	19.4	66	27.3
Current age(n, mean, sd)	302	29.3(3.5)	361	28.8(3.7)	201	30.1(3.9)	242	29.3(3.7)
Gender								
Male	135	44.7	140	38.8	89	44.3	87	36.0
Female	167	55.3	221	61.2	112	55.7	155	64.0
% White race	298	98.7	353	97.8	201	100.0	237	97.9
Current Educational Level								
High school graduate or less	39	12.9	55	15.2	18	9.0	30	12.4
Some college	63	20.9	75	20.8	43	21.4	70	28.9
Four year college grad or higher	200	66.2	231	64.0	140	69.7	141	58.3
Missing	0	0.0	0	0.0	0	0.0	1	0.4
Current marital status								
Single	84	27.8	134	37.1	60	29.9	68	28.1
Married or cohabitating	211	69.9	220	60.9	134	66.7	166	68.6
Other	6	2.0	4	1.1	5	2.5	5	2.1
Missing	1	0.3	3	0.8	2	1.0	3	1.2
Mother’s age at subject’s birth (n, mean (sd))	302	27.9 (4.7)	361	27.1 (4.5)	201	28.0 (4.5)	242	27.1 (4.3)
Father’s age at subject’s birth (n, mean (sd))	302	30.8 (6.1)	361	29.5 (5.3)	201	30.7 (5.4)	241	29.3 (4.7)
Mother’s educational level at subject’s birth								
High school graduate or less	64	21.2	91	25.2	28	13.9	51	21.1
Some college	80	26.5	135	37.4	66	32.8	94	38.8
Four year college grad or higher	156	51.7	131	36.3	105	52.2	96	39.7
Missing	2	0.7	4	1.1	2	1.0	1	0.4
Father’s occupation at subject’s birth								
White collar	172	57.0	183	50.7	109	54.2	110	45.5
Blue collar	91	30.1	114	31.6	57	28.4	71	29.3
Other	37	12.3	58	16.1	32	15.9	58	24.0
Missing	2	0.7	6	1.7	3	1.5	3	1.2
Subject breast fed								
Yes	194	64.2	209	57.9	133	66.2	165	68.2
No	102	33.8	148	41.0	62	30.8	77	31.8
Missing	6	2.0	4	1.1	6	3.0	0	0.0
Number of older siblings								
0	133	44.0	148	41.0	97	48.3	116	47.9
1	94	31.1	142	39.3	59	29.4	78	32.2
2+	75	24.8	71	19.7	45	22.4	48	19.8
Mother’s cigarette smoking during subject’s gestation								
11+ cigarettes a day	62	20.5	46	12.7	33	16.4	26	10.7
10 or fewer cigarettes a day	44	14.6	30	8.3	34	16.9	20	8.3
None	195	64.6	285	78.9	134	66.7	196	81.0
Missing	1	0.3	0	0.0	0	0.0	0	0.0
Mother’s alcohol consumption during subject’s gestation								
1+ drinks a week	109	36.1	–	–	76	37.8	–	–
1–3 drinks a month	193	63.9	–	–	125	62.2	–	–
None	–	–	361	100.0	–	–	242	100.0
Missing								
Mother’s use of marijuana during subject’s gestation								
Yes	20	6.6	5	1.4	15	7.5	3	1.2
No	281	93.0	356	98.6	182	90.5	238	98.3
Missing	1	0.3	0	0.0	4	2.0	1	0.4

^a^Excludes all subjects missing prenatal alcohol data

The results for teenage alcohol use and early life exposure to PCE and alcohol indicated little increased risk with exposure (Table 2). For early age at first consumption of alcoholic beverages (<=13 years vs. 14+ years old), there was very little evidence of increased risk for combined exposure to PCE and alcohol (RR = 1.3, 95% CI 0.9, 1.9) There was also little effect for each exposure individually. Results were similar for frequent drinking as a teenager among subjects with combined exposure to PCE and alcohol and PCE exposure alone.

**Table 2:** Teenage Alcohol Use and Early Life Exposure to PCE and Alcohol

Outcome	Combined Exposure	% Yes (n/N)	Crude RR (95% CI)	Simple GEE RR (95% CI)
First drank at ≤13 years vs. 14+ years	Any PCE/Any alcohol	22.3 (67/301)	1.4 (0.9,1.9)	1.3 (0.9,1.9)
	Any PCE/No alcohol	17.9 (62/346)	1.1 (0.8,1.6)	1.1 (0.7,1.6)
	No PCE/Any alcohol	18.0 (35/194)	1.1 (0.7,1.7)	1.1 (0.7,1.7)
	No PCE/No alcohol	16.4 (38/232)	Reference	Reference
Drank >8 days/mo as teen vs. Never drank as teen	Any PCE/Any alcohol	27.6 (24/87)	1.4 (0.8,2.4)	1.3 (0.8,2.3)
	Any PCE/No alcohol	28.9 (35/121)	1.5 (0.9,2.4)	1.4 (0.9,2.3)
	No PCE/Any alcohol	23.4 (11/47)	1.2 (0.6,2.3)	1.1 (0.5,2.2)
	No PCE/No alcohol	19.6 (19/97)	Reference	Reference
Drank ≥5/4 drinks/day as a teen vs. Never drank as a teen	Any PCE/Any alcohol	63.8 (111/174)	1.4 (1.2,1.8)	1.4 (1.1,1.7)
	Any PCE/No alcohol	57.4 (116/202)	1.3 (1.0,1.6)	1.3 (1.0,1.6)
	No PCE/Any alcohol	66.4 (71/107)	1.5 (1.2,1.9)	1.4 (1.1,1.8)
	No PCE/No alcohol	44.3 (62/140)	Reference	Reference

Specific drugs were examined including crack/cocaine, psychedelics/hallucinogens, club/designer drugs, Ritalin without a prescription, heroin and marijuana. Many subjects had co-occurring drug use and so when drugs were combined into categories for analysis, small increased risks for using two or more drugs as a teen were observed among individuals exposed to PCE and alcohol individually (RRs: 1.2–1.3, Table 3). This risk was slightly higher for individuals with both exposures (RR:1.5). Similar results were observed for using any major drugs. The greatest increased risk was observed for using two or more major drugs, where the effect of PCE and alcohol exposure together was greater (RR = 1.9 95% CI 1.2, 3.0) than the effect of PCE exposure (RR = 1.6, 95% CI 1.0, 2.4) and alcohol (RR = 1.3, 95% CI 0.7, 2.1) alone. The most commonly used drug was marijuana, although there was little effect for ever using marijuana among individuals exposed to both alcohol and PCE exposure (RR = 1.2, 95% CI 1.1, 1.4). Numbers of subjects were small for other drugs, particularly heroin (*n* = 8), but the results did consistently demonstrate increased risk among individuals exposed to both PCE and alcohol (Table 4).

**Table 3:** Teenage Drug Use by Category and Early Life Exposure to PCE and Alcohol

Outcome	Combined Exposure	% Yes (n/N)	Crude RR (95% CI)	Simple GEE RR (95% CI)
Used any drugs as teen vs never used any drugs	Any PCE/Any alcohol	72.3 (180/249)	1.3 (1.1,1.4)	1.2 (1.1,1.4)
	Any PCE/No alcohol	62.0 (183/295)	1.1 (0.9,1.2)	1.1 (0.9,1.3)
	No PCE/Any alcohol	67.3 (111/165)	1.2 (1.0,1.4)	1.2 (1.0,1.4)
	No PCE/No alcohol	57.6 (117/203)	Reference	Reference
Used 2 or more drugs as teen vs never used any drugs	Any PCE/Any alcohol	54.3 (82/151)	1.6 (1.2,2.1)	1.5 (1.1,2.0)
	Any PCE/No alcohol	44.3 (89/201)	1.3 (1.0,1.7)	1.3 (0.9,1.7)
	No PCE/Any alcohol	43.2 (41/95)	1.3 (0.9,1.7)	1.2 (0.9,1.7)
	No PCE/No alcohol	34.4 (45/131)	Reference	Reference
Used major drugs (excl. marijuana) as teen vs never used any drugs	Any PCE/Any alcohol	54.9 (84/153)	1.6 (1.2,2.1)	1.5 (1.2,2.1)
	Any PCE/No alcohol	45.1 (92/204)	1.3 (1.0,1.7)	1.3 (1.0,1.7)
	No PCE/Any alcohol	44.3 (43/97)	1.3 (0.9,1.8)	1.2 (0.9,1.7)
	No PCE/No alcohol	34.4 (45/131)	Reference	Reference
Used 2 or more major drugs as teen vs never used any drugs	Any PCE/Any alcohol	40.0 (46/115)	2.0 (1.3,3.0)	1.9 (1.2,3.0)
	Any PCE/No alcohol	31.7 (52/164)	1.6 (1.0,2.4)	1.6 (1.0,2.4)
	No PCE/Any alcohol	26.0 (19/73)	1.3 (0.7,2.2)	1.3 (0.7,2.1)
	No PCE/No alcohol	20.4 (22/108)	Reference	Reference

**Table 4:** Teenage Drug Use by Type and Early Life Exposure to PCE and Alcohol

Outcome	Combined Exposure	% Yes (n/N)	Crude RR (95% CI)	Simple GEE RR (95% CI)
Used marijuana as teen vs never used any drugs	Any PCE/Any alcohol	72.2 (179/248)	1.3 (1.1,1.4)	1.2 (1.1,1.4)
	Any PCE/No alcohol	61.5 (179/291)	1.1 (0.9,1.2)	1.1 (0.9,1.2)
	No PCE/Any alcohol	66.9 (109/163)	1.2 (1.0,1.4)	1.1 (1.0,1.4)
	No PCE/No Alcohol	57.6 (117/203)	Reference	Reference
Used inhalants as teen vs never used any drugs	Any PCE/Any alcohol	22.5 (20/89)	1.7 (0.9,3.2)	1.7 (0.9,3.3)
	Any PCE/No alcohol	18.2 (25/137)	1.4 (0.7,2.6)	1.4 (0.7,2.6)
	No PCE/Any alcohol	18.2 (12/66)	1.4 (0.7,2.8)	1.4 (0.7,2.9)
	No PCE/No Alcohol	13.1 (13/99)	Reference	Reference
Used heroin as teen vs never used any drugs	Any PCE/Any alcohol	4.2 (3/72)	1.8 (0.3, 11)	1.8 (0.3, 11)
	Any PCE/No alcohol	1.8 (2/114)	0.8 (0.1,5.4)	0.8 (0.1,5.4)
	No PCE/Any alcohol	1.8 (1/55)	0.8 (0.1,8.6)	0.8 (0.1,8.6)
	No PCE/No Alcohol	2.3 (2/88)	Reference	Reference
Used cocaine/crack as teen vs never used any drugs	Any PCE/Any alcohol	33.0 (34/103)	2.4 (1.3,4.1)	2.3 (1.3,4.1)
	Any PCE/No alcohol	25.3 (38/150)	1.8 (1.0,3.2)	1.8 (1.0,3.2)
	No PCE/Any alcohol	15.6 (10/64)	1.1 (0.5,2.4)	1.1 (0.5,2.4)
	No PCE/No Alcohol	14.0 (14/100)	Reference	Reference
Used psychedelics/hallucinogens as teen vs never used any drugs	Any PCE/Any alcohol	46.5 (60/129)	1.9 (1.3,2.8)	1.9 (1.3,2.8)
	Any PCE/No alcohol	37.8 (68/180)	1.6 (1.1,2.3)	1.6 (1.1,2.3)
	No PCE/Any alcohol	37.9 (33/87)	1.6 (1.0,2.4)	1.6 (1.0,2.4)
	No PCE/No Alcohol	23.9 (27/113)	Reference	Reference
Used ritalin without prescription as teen vs never used any drugs	Any PCE/Any alcohol	28.9 (28/97)	2.4 (1.3,4.4)	2.2 (1.1,4.0)
	Any PCE/No alcohol	18.8 (26/138)	1.5 (0.8,2.9)	1.5 (0.8,2.8)
	No PCE/Any alcohol	16.9 (11/65)	1.4 (0.6,2.9)	1.3 (0.6,2.8)
	No PCE/No Alcohol	12.2 (12/98)	Reference	Reference
Used club drugs/designer drugs as teen vs never used any drugs	Any PCE/Any alcohol	28.9 (28/97)	1.7 (1.0,2.8)	1.7 (1.0,2.9)
	Any PCE/No alcohol	26.8 (41/153)	1.5 (0.9,2.5)	1.6 (0.9,2.6)
	No PCE/Any alcohol	16.9 (11/65)	1.0 (0.5,1.9)	1.0 (0.5,2.0)
	No PCE/No Alcohol	17.3 (18/104)	Reference	Reference

## Discussion

In this exploratory analysis of a retrospective birth cohort from Cape Cod, Massachusetts we found evidence of independent effects on teenage illicit drug and alcohol use in relation to early life exposures to PCE and alcohol. When both exposures were present, the effect on illicit drug use appeared to be additive, particularly in the instance of using two or more major drugs. More modest increases were seen in alcohol use as a teenage with early life exposure to both PCE and alcohol.

The analyses accounted for available confounding variables, including the child’s history of learning disabilities and mental illness, family history of mental illness, and maternal behaviors during pregnancy such as cigarette smoking and marijuana use [29–33]. In addition, previous analyses of this population indicated PCE exposure was not associated with learning disabilities [34], early puberty [34] or depression [35], possible intermediates to risk-taking behaviors. There was some support of an impact of PCE on other mental health outcomes, bipolar disorder (*n* = 31) and post-traumatic stress disorder (*n* = 39), but the number of subjects affected in our study population was small [35] and excluding these subjects from the analysis did not alter the results.

These results support the hypothesis of similar behavioral effects of these two solvents but also highlight the challenges of conducting research on the long-term behavioral effects of early life exposures. Risk-taking behaviors occurred many years after the early life exposures and while we were able to account for many confounding variables, we were unable to take into account important social risk factors present during childhood and adolescence. These included level of parental supervision; parental behaviors including drug and alcohol use; family relationships and occurrence of abuse, neglect and violence; and peer and neighborhood influences [29–33]. Information on these sensitive topics was not obtained because of their complicated nature and the likelihood of poor recall by now adult subjects.

Other study imitations include potential for exposure misclassification. For PCE, we used a modeled rather than measured exposure to PCE in drinking water. In addition to assuming all residences were occupied and used the same amount of water, the water model used a representative set of pipe and water source conditions from 1980 and applied them to entire study period from 1969–1983. The limited historical water sampling data from that time have demonstrated moderate correlation between modeled and sampled values [36, 37]. For prenatal alcohol use and outcome measures of alcohol and illicit drug use, the study results may be impacted by retrospective self-reports. Subjects were between 25 and 40 years old when they were asked to recall behaviors from their teenage years. Prenatal alcohol exposure may also have been underreported as the consumption of alcohol while pregnant is stigmatized and subjects were recalling pregnancies that were 20 to 35 years prior. However, given the historical study period, we believe that this was more likely to affect the self-reported amount and frequency of alcohol use, rather than the report of ever consumption. The number of subjects was too small in each PCE-alcohol exposure category to allow examination by frequency of prenatal alcohol consumption. There may also have been under-reporting of the risk-taking behaviors, due to recall problems or an aversion to report drug or alcohol use, although surveys have shown similar prevalence of these behaviors in the same geographic area [38, 39].

Selection bias does not appear to have affected these results, although the low response rate does require comment. A comparison of non-participants and participants indicated that even though participants were more likely to be female and have college educated mothers, this was evident for both PCE exposed and unexposed subjects [1]. We cannot examine differences between participants and non-participants by prenatal alcohol since this was determined by survey whereas PCE exposure status was available from the residence listed on the birth certificate. Loss of potential subjects to death was small and unrelated to PCE exposure status. Death due to substance use was determined to affect only four subjects according to Massachusetts Vital Records [1].

Numerous animal and epidemiological studies have demonstrated neurotoxicity of PCE [3, 40] and prenatal alcohol exposure [18]. Consistent findings for PCE exposure were primarily in vision, visuospatial memory and neuropsychological function [3]. The relationship of prenatal alcohol exposure and intellectual disability is known, with a few studies noting social behavior deficits in humans with support from basic animal research [41]. Consistent findings for PCE are primarily for adult exposure, while studies of in utero exposure to PCE have had varied results. A small study of children with mothers with occupational solvent exposure did not find any associations with measures of intellectual ability, growth, development or behavior [42], while two other also small scale studies found lower test scores for intellectual, language, motor, and neurobehavioral functioning [12, 13]. No effect on cognition, behavior or learning and attention disorders was observed in other studies of exposure to PCE at a day care and preschool located near a dry cleaning establishment [43, 44] or from contaminated drinking water contaminated [34].

Our findings are consistent with studies showing lasting effects of prenatal alcohol exposure on alcohol use in offspring. A study in two Seattle, Washington clinics of over 1,500 women used self-reported data on alcohol use collected mid-pregnancy and found that prenatal alcohol exposure was significantly associated with alcohol problems in their offspring at 21 years of age [15]. However, it is unclear if these results are due to alcohol exposure itself or to the social environment of having a parent who drinks. Similarly, an Australian population-based birth cohort study found that higher prenatal alcohol exposure increased the risk of developing early onset alcohol disorders at age 21, with higher risks observed for exposures during early pregnancy (OR = 2.95, 95% CI 1.62–5.36) than later pregnancy (OR = 1.35, 95% CI 0.69–2.63) [14]. Additionally, basic research in animals has indicated prenatal alcohol exposure influences later postnatal response to the odor and taste of alcohol [16]. A small study found higher relative rates of “pleasantness” for alcohol in young people with prenatal exposure to alcohol [45]. It may be that a later preference for the odor and taste for alcohol is the result of these similar stimuli from early exposures [19].

There is little comparable research on maternal alcohol exposure and drug use in their offspring. One recent study of 5,922 children and adolescents in Germany observed that low to moderate prenatal alcohol exposure was associated with an increased risk of ever using illicit drugs (OR = 1.62, 95% CI 1.23, 2.14) [46]. When stratified by gender, stronger associations were observed for females but there was little association for males. Notably, we observed few differences when we stratified by gender. Another small study also observed some influence on later drug use disorders in a high-risk group of offspring from families with a high density of alcohol dependence [47]. A high prevalence drug use (29%) as teenagers has been reported among a group of subjects diagnosed with fetal alcohol syndrome and fetal alcohol effects in Washington state [48].

We found evidence for an additive effect of early life exposure to PCE and alcohol on the risk of multiple illicit drug use as a teenager. By additive, we mean the epidemiologic definition that the impact of combined exposure is equal to sum of the impacts of each exposure alone [49]. It is worth noting that epidemiologists and toxicologists use different definitions of additivity and interaction, although they coincide when dose-response functions are linear [50]. Since PCE and alcohol are both solvents it is plausible that they act by similar mechanisms on the behaviors under study. Because of the categorization of data on prenatal alcohol exposure and small number of subjects, we cannot examine additivity using appropriate toxicology-based methods.

## Conclusions

Environmental, biological and social factors likely influence the initiation of risk-taking behaviors such as illicit drug and alcohol use. This exploratory analysis provides support for an additive effect of early life exposure to PCE and alcohol on the risk of use of multiple illicit drugs as a teenager. Because of numerous limitations in this retrospective study, further research is needed to examine longstanding behavioral effects of early life exposures. To be most informative, this research should involve detailed prospective data collection.

## Abbreviations

CI: Confidence interval
FASD: Fetal alcohol spectrum disorders
GEE: Generalized estimating equation
PCE: Tetrachloroethylene
RR: Risk ratio
USEPA: United States Environmental Protection Agency
VL/AC: Vinyl-lined asbestos-cement
